# Microbial signatures in metastatic cancer

**DOI:** 10.3389/fmed.2025.1654792

**Published:** 2025-09-30

**Authors:** Jhommara Bautista, María Paula Fuentes-Yépez, Joseth Adatty-Molina, Andrés López-Cortés

**Affiliations:** ^1^Cancer Research Group (CRG), Faculty of Medicine, Universidad de Las Américas, Quito, Ecuador; ^2^Faculty of Engineering, Heidelberg University, Heidelberg, Germany; ^3^Hospital General Universitario Dr. Balmis, Alicante, Spain

**Keywords:** microbial signatures, metastatic cancer, microbiome, metastasis, tumor microenvironment, immunoediting, intratumoral bacteria, spatial-multi-omic approaches

## Abstract

Metastasis remains the leading cause of cancer-related death, yet the biological determinants that enable tumor cells to disseminate and colonize distant organs are incompletely understood. Emerging evidence identifies the microbiome, not merely as a bystander, but as an active architect of the metastatic cascade. Microbial communities residing in the gut, mucosal barriers, and within tumors shape metastatic progression by modulating immune surveillance, stromal remodeling, oncogenic signaling, and therapy response. Intratumoral and even intracellular microbes regulate epithelial–mesenchymal transition, angiogenesis, and immune escape, while gut-derived metabolites condition pre-metastatic niches and alter systemic immunity. Technological advances in spatial transcriptomics, single-cell multi-omics, and metagenomics have revealed a spatially organized, functionally integrated microbial ecosystem within tumors, challenging long-held assumptions of sterility in cancer biology. This review synthesizes five converging dimensions of this paradigm: microbial interactions in the metastatic tumor microenvironment; microbiome-mediated immunoediting and metastatic escape; the role of intratumoral and intracellular bacteria in dissemination; spatial-multi-omic approaches to map microbial niches; and microbial biomarkers predictive of metastasis and therapy outcomes. Collectively, these findings recast the microbiome as a critical and targetable determinant of metastasis. Deciphering the tumor–microbe–host triad holds transformative potential for biomarker development, therapeutic innovation, and precision oncology.

## Introduction

Metastasis, the dissemination of cancer cells from a primary tumor to distant organs, accounts for the vast majority of cancer-related deaths. While once attributed solely to intrinsic tumor cell properties and host immune responses, it is now evident that the microbiome plays a fundamental role in shaping the metastatic cascade. Microbial communities residing in the gut, at mucosal barriers, and within tumors interact with cancer and immune cells through diverse molecular and metabolic pathways, influencing invasion, immune editing, stromal remodeling, and therapy resistance (1–3). Historically considered sterile, tumors are now recognized to harbor a rich and functionally active microbiome. Advances in high-throughput sequencing, spatial transcriptomics, and single-cell analysis have uncovered that both commensal and pathogenic microorganisms, including bacteria, fungi, and viruses, are present within the tumor microenvironment, sometimes even within cancer cells themselves (4–6). These intratumoral and intracellular microbes are not passive bystanders; they actively regulate oncogenic signaling, epithelial-mesenchymal transition (EMT), angiogenesis, and immune surveillance. For example, intracellular bacteria have been shown to modulate tumor cell contractility, promoting intravasation and dissemination without affecting primary tumor growth (5, 7).

Beyond local effects, the gut microbiota exerts a systemic influence over distant metastatic niches. Microbial metabolites such as short-chain fatty acids (SCFAs), bile acids, and tryptophan derivatives can modulate immune tone, endothelial permeability, and stromal cell activation at distal organs, predisposing them to metastatic colonization (2, 3, 8). Additionally, gut dysbiosis induced by diet, antibiotics, or prior therapy has been linked to resistance to immune checkpoint inhibitors (ICIs) and reduced survival in multiple cancer types (1, 9). Conversely, specific microbial signatures are associated with improved response to immunotherapy and chemotherapy, suggesting that the microbiome may be harnessed to enhance therapeutic efficacy (6, 10). The emerging concept of the “microbial tumor ecosystem” positions microbes as key modulators of metastatic behavior. This review examines five interconnected dimensions of this paradigm: (1) microbiome interactions in metastatic tumor microenvironments, (2) microbiome-mediated immunoediting and metastatic escape, (3) the role of intratumoral and intracellular bacteria in promoting dissemination, (4) spatial and multi-omic approaches for mapping microbial tumor interactions, and (5) microbial biomarkers that predict metastasis and therapy outcomes. Together, these perspectives highlight a growing recognition that microbial communities are not ancillary to cancer but integral to its progression and potentially, its control.

## Microbiome interactions in metastatic tumor microenvironments

**FIGURE 1:**
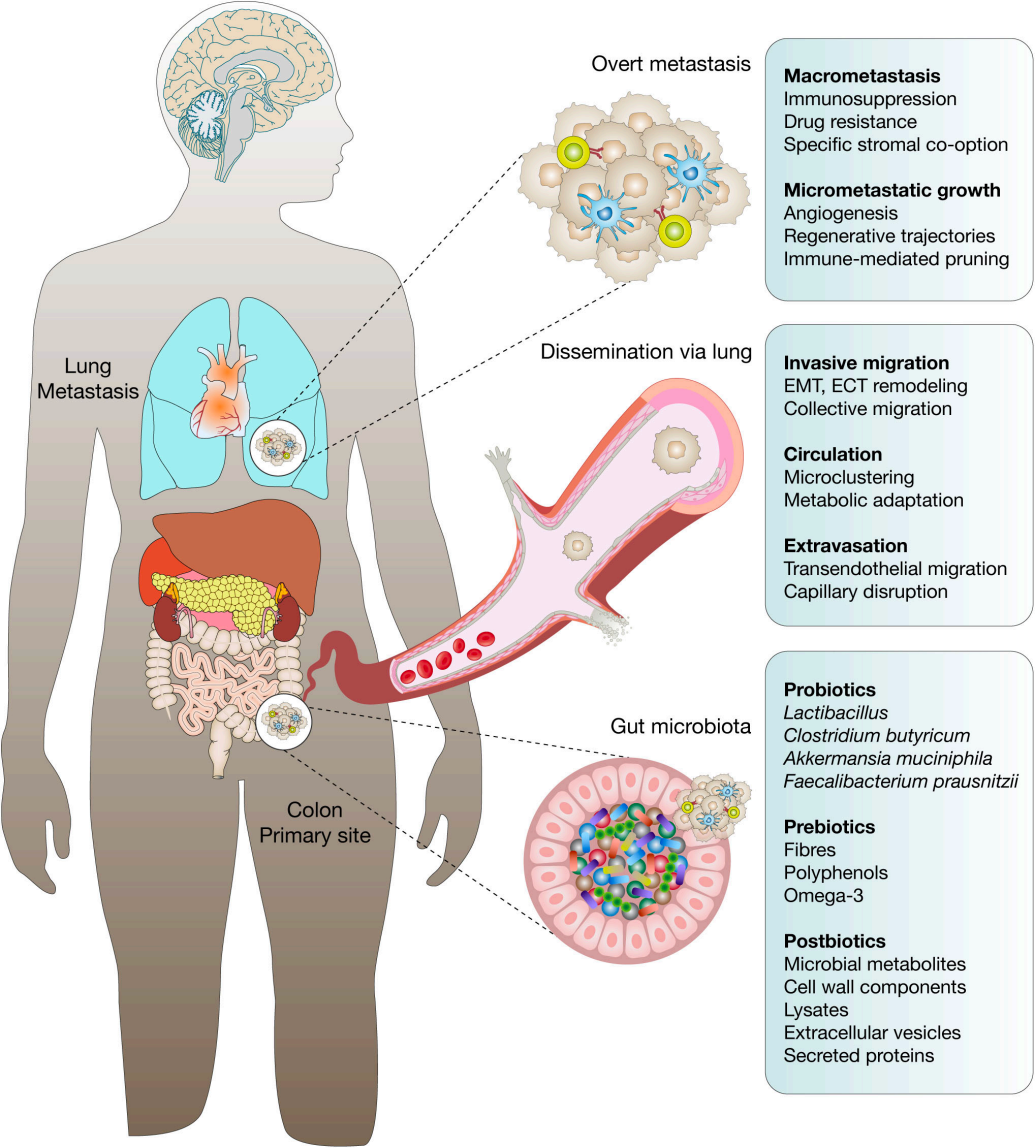
Microbial signatures in metastatic cancer progression. This schematic illustrates the dynamic influence of the microbiome and its derivatives, probiotics, prebiotics, and postbiotics, across distinct phases of metastasis originating from a primary colorectal tumor. In the healthy gut, beneficial microbial taxa, including *Lactobacillus*, *Clostridium butyricum*, *Akkermansia muciniphila*, and *Faecalibacterium prausnitzii*, are supported by prebiotic substrates such as dietary fibers, polyphenols, and omega-3 fatty acids. These communities generate postbiotics, including microbial metabolites, cell wall components, lysates, extracellular vesicles, and secreted proteins, that modulate host immunity, barrier integrity, and metabolic tone. Upon oncogenic transformation, microbial dysbiosis may contribute to metastatic initiation. At the primary site, tumor cells undergo epithelial–mesenchymal transition (EMT), extracellular matrix remodeling, and collective migration to invade surrounding tissue. Dissemination occurs via hematogenous spread, with tumor cells forming microclusters and undergoing metabolic adaptation in circulation. In the lungs, micrometastatic niches are characterized by angiogenesis, regenerative-like transcriptional trajectories, and immune-mediated pruning. Progression to overt macrometastases involves stromal co-option, immune suppression, and therapy resistance. The figure integrates microbiome-derived factors that influence each metastatic stage, emphasizing the therapeutic potential of microbiome modulation to disrupt metastatic cascades and restore immune competence.

The tumor microenvironment (TME) functions as a highly dynamic and complex ecosystem shaped by cancer cells, stromal components, and increasingly, microbial communities that either reside within tumors or interact with them from distant niches such as the gut. Recent studies have established that both gut-derived and intratumoral microbes can actively modulate metastatic progression by influencing local immune responses, remodeling the extracellular matrix (ECM), and altering signaling within metastatic niches (11, 12). Microbial colonization of metastatic sites has been consistently observed across cancer types, with pan-cancer analyses detecting bacterial DNA in over 4,000 metastatic biopsies (7). These microbes exhibit organ-specific tropism, with hypoxic environments showing enrichment for specific taxa, and a strong association has been noted between microbial diversity and neutrophil infiltration, as well as resistance to ICIs, particularly in non-small cell lung cancer (9, 13). Tumor-associated microbes are not passive elements; rather, they engage in metabolic and immunological crosstalk with host cells, regulating immune surveillance, influencing drug bioavailability, and promoting metastasis via processes such as EMT and formation of pre-metastatic niches (14). Certain bacteria degrade ECM components and facilitate tumor invasion while simultaneously inducing chronic inflammation that prepares distant sites for colonization and contributes to immune evasion (15). Tumors may also shape their microbial communities to enhance tumorigenesis and metastatic fitness through inflammation, metabolic adaptation, and immune modulation (13, 16). Microbiota-derived metabolites like SCFAs, indoles, and bile acid derivatives exert profound effects on angiogenesis, stromal activation, and immune cell recruitment; their imbalance due to dysbiosis can favor metastasis through accumulation of genotoxic and pro-inflammatory species (11). Moreover, gut dysbiosis has been implicated in diminished ICI responses, while restoring beneficial microbial taxa has been shown to reestablish antitumor immunity, particularly in melanoma and Non-Small Cell Lung Cancer (NSCLC) (12). Mechanistically, microbial interactions influence key aspects of the TME such as metabolic reprogramming, stromal remodeling, and immune cell dynamics (9). Single-cell transcriptomics has revealed that gut microbiota modulate tumor-associated macrophage phenotypes, promoting the conversion of immunosuppressive Spp1 + TAMs into antigen-presenting CD74 + macrophages and enhancing CD8 + T cell responses via γδ T cell–mediated CD40L signaling (17). The metastatic TME evolves through paracrine, contact-dependent, and vesicle-mediated signaling between host cells and microbes, with microbial signals tailoring stromal behavior and immune tolerance in a context-specific manner (16). Tumor-resident microbes (TRM), distinct from transient microbiota, persist within tumors and significantly affect cellular signaling, immune infiltration, and therapeutic outcomes across both primary and metastatic lesions (15, 18). Collectively, these findings redefine the microbiome as a pivotal component of the metastatic niche, offering novel insights into metastatic pathophysiology and pointing to microbial signatures and functions as promising biomarkers and therapeutic targets for metastatic cancer (Figure 1).

## Microbiome-mediated immunoediting and metastatic escape

The microbiome is increasingly recognized as a key modulator of cancer immunoediting, the dynamic process by which the immune system shapes tumor evolution through phases of elimination, equilibrium, and escape. Within this framework, microbial communities can influence tumor immune visibility, editing of neoantigen profiles, and the efficacy of antitumor immunity (19, 20). Dysbiosis alters immune cell recruitment and polarization, enabling malignant cells to evade immune detection and colonize distant organs. The gut microbiota regulates innate and adaptive immune responses by modulating cytokine production, antigen presentation, and costimulatory signals, thereby shaping tumor-immune interactions that influence metastatic escape (21, 22). Certain microbial taxa promote the expansion of regulatory T cells and myeloid-derived suppressor cells while impairing antigen-presenting cell function, creating immunosuppressive microenvironments that facilitate metastatic dissemination. Microbial metabolites such as SCFAs, polyamines, and tryptophan catabolites can suppress antitumor immunity and promote immune tolerance in metastatic niches (23, 24).

Emerging studies reveal that microbial composition can predict response to ICI, with distinct microbial signatures correlating with durable response, progression-free survival, and immune-related toxicities. Importantly, the microbiome influences ICI-mediated immunoediting by modulating dendritic cell maturation, IFN-γ signaling, and cytotoxic T cell infiltration (21, 25). For instance, strain-level resolution of gut microbes improved prediction of clinical responses to anti-PD-1/CTLA-4 therapies across cancer types, suggesting that microbial immunomodulation contributes directly to metastatic immune escape or containment (20, 26). In colorectal cancer, microbial-induced epigenetic and transcriptomic changes contribute to immune exclusion, and gut-derived *Fusobacterium nucleatum* promotes resistance to T cell-mediated killing by downregulating MHC class I and activating autophagy-dependent survival pathways (19).

Mechanistically, microbes act as both immunological triggers and shields. They regulate immune editing by influencing T cell repertoire diversity, priming or depleting tumor reactive clones, and reprogramming antigen presentation pathways (23). Some commensals promote cross-presentation and clonal expansion of effector T cells, while others enhance tumor immune escape via modulation of type I interferon responses or STING pathway suppression (27, 28). The cancer-microbiome-immune axis extends beyond local effects to systemic immune modulation, with microbiota-derived signals propagating through metabolites, extracellular vesicles, and microbial-associated molecular patterns (MAMPs) to condition peripheral immune responses and pre-metastatic sites (25, 26). Altogether, these findings support the paradigm that the microbiome is a critical architect of immunoediting and metastatic immune evasion. Deciphering the microbial determinants of immune escape not only elucidates fundamental mechanisms of metastasis but also opens new avenues for biomarker development and microbiota-informed immunotherapeutic strategies.

## Intratumoral and intracellular bacteria and their impact on metastatic behavior

Intratumoral and intracellular bacteria have emerged as active participants in cancer progression, exhibiting organ-specific colonization patterns and influencing key hallmarks of metastasis (29, 30). These bacteria are not passive passengers but functional constituents of the tumor microenvironment, capable of modulating oncogenic signaling, DNA integrity, immune surveillance, and therapeutic resistance. Recent studies have demonstrated the presence of viable bacteria within the cytoplasm of tumor cells across multiple cancer types, including breast, lung, and pancreatic tumors. These intracellular microbes evade conventional antibiotics, manipulate host signaling pathways, and persist under hypoxic and immunosuppressive conditions within the tumor microenvironment (4, 31, 32). The detection of bacteria in tumors once considered sterile, such as brain or bone metastases, challenges conventional paradigms and highlights the importance of re-evaluating microbial contributions to metastasis (33). Intratumoral microbes can induce EMT, promote angiogenesis, and facilitate extracellular matrix remodeling, all of which are critical to metastatic dissemination. They modulate host cell metabolism and epigenetics, triggering pro-metastatic programs through reactive oxygen species (ROS) production, DNA damage, and altered chromatin landscapes (1, 30, 34). These changes can promote tumor cell survival in circulation, support immune evasion, and enhance colonization at distant sites.

Bacteria residing within tumor cells also alter responses to chemotherapy by degrading drugs, interfering with apoptotic signaling, or shifting the balance toward autophagy-mediated survival. For example, cytidine deaminase-expressing bacteria have been shown to metabolize gemcitabine, reducing its cytotoxicity in pancreatic cancer models (29, 33). Moreover, intratumoral bacteria have been associated with differential immune infiltration, often favoring immunosuppressive phenotypes dominated by myeloid-derived suppressor cells and alternatively activated macrophages. These immune deviations contribute to the formation of metastatic niches that are tolerant to immune surveillance and primed for tumor expansion (1, 35). Technological advances in metagenomic sequencing, *in situ* hybridization, and high-resolution microscopy have revealed the spatial localization and intracellular residency of these bacteria, affirming their presence and function even in low-biomass environments. Functional studies in germ-free mice, patient-derived xenografts, and organoid models further confirm their causative role in modulating tumor progression (25, 36). Emerging evidence suggests that bacteria can disseminate with tumor cells, co-migrating during metastasis and contributing to metastatic niche conditioning (4). The coevolution of cancer cells and associated microbes within the tumor ecosystem is increasingly appreciated as a driver of metastatic behavior (31, 34). In summary, intratumoral and intracellular bacteria reshape the metastatic trajectory of cancer by promoting immune evasion, altering therapy response, and activating pro-metastatic signaling. Their functional integration into tumor biology suggests they are not merely diagnostic curiosities but potential therapeutic targets and prognostic markers in metastatic disease.

## Integrating spatial and multi-omic approaches to map microbial tumor ecosystems

Mapping the spatial architecture and molecular complexity of microbial tumor ecosystems requires integrative frameworks that transcend traditional bulk analyses (37). Spatial and multi-omic technologies have revolutionized our understanding of how tumor cells, immune constituents, stromal populations, and resident microbes interact across tissue landscapes (38). These approaches preserve spatial context while decoding genetic, transcriptomic, proteomic, metabolomic, and microbial profiles at single-cell and subcellular resolution, providing unprecedented insight into tumor heterogeneity and microbe-host interplay (37, 39, 40). Spatial transcriptomics, especially when integrated with single-cell RNA sequencing, enables reconstruction of the tumor-microbiome landscape with spatial fidelity. These methods reveal that intratumoral microbial communities are not randomly distributed but localize to specific tumor niches, such as hypoxic zones, invasive fronts, and immune-excluded regions. Microbial-immune crosstalk is spatially constrained, with microbial hubs often colocalizing with immunosuppressive myeloid clusters or fibrotic stromal regions. Computational frameworks like Cottrazm leverage spatially resolved transcriptomics and histology to delineate tumor boundaries and identify cell-type-specific gene expression at the tumor-host interface, uncovering how microbial proximity shapes immune exclusion and T cell infiltration barriers (37, 41, 42).

Spatial multi-omics also supports the identification of microbial metabolites and bacterial RNA signatures embedded in tissue sections, illuminating metabolic exchange between microbes and host cells. Co-detection of microbial transcripts alongside host cell states reveals transcriptional reprogramming of immune cells near microbial niches, including enhanced expression of immune checkpoints, altered antigen presentation capacity, and cytokine signatures indicative of immune tolerance. These fine-scale interactions underscore how spatially anchored microbial signals contribute to shaping immune gradients across tumors and may condition pre-metastatic niches at distant sites (38, 43, 44). Technologies such as MERFISH, CosMx, Slide-seq, and 10x Genomics Visium allow multiplexed profiling of microbial host interactions with subcellular precision (44). While spacecraft-like technologies (e.g., LCM and targeted ROI profiling) excel in dissecting localized features of microbe-enriched tumor regions, telescope-like spatial landscaping platforms enable a panoramic view of microbial host dynamics across entire tumor sections. This dual-scale strategy enhances resolution while capturing ecosystem-wide patterns that govern tumor-microbe coevolution (45). Integrating spatial data with metagenomics, metabolomics, and epigenomics provides a multilayered map of the tumor-microbiome (39). This systems-level approach reveals how microbial presence influences chromatin accessibility, transcription factor binding, and metabolic flux in adjacent host cells. Spatially-aware machine learning algorithms now allow for the prediction of microbial niches, immune landscapes, and therapeutic response signatures based on multimodal input, setting the stage for microbiome-informed precision oncology (37, 42). In summary, the convergence of spatial and multi-omic platforms has transformed our capacity to decode the structure, function, and influence of microbial tumor ecosystems. These integrative strategies are illuminating microbial determinants of tumor behavior, uncovering spatial biomarkers of metastasis, and identifying novel targets for intervention. As spatial technologies continue to evolve, their application to microbial tumor ecology holds promise for the development of spatially resolved microbiome-based diagnostics and therapeutics in metastatic cancer.

## Microbial biomarkers for predicting metastasis and therapy outcomes

Microbial biomarkers are emerging as powerful tools for predicting cancer metastasis, therapy response, and clinical outcomes, particularly in the context of immunotherapy and precision oncology (46). These biomarkers include specific bacterial taxa, microbial gene signatures, circulating microbial DNA (cmDNA), and bacterial metabolites, all of which can reflect or modulate tumor progression and treatment efficacy across multiple cancer types (24, 47). Multiple studies have identified distinct microbial profiles associated with metastatic risk. For example, the enrichment of *Fusobacterium nucleatum* has been consistently associated with colorectal cancer metastasis and poor prognosis, while elevated levels of *Akkermansia muciniphila* correlate with enhanced immune infiltration and response to checkpoint inhibitors in lung and melanoma patients (48, 49). Specific bacteria, including *Bacteroides fragilis, Bifidobacterium longum*, and *Enterococcus hirae*, have been linked to durable responses to PD-1 and CTLA-4 blockade, demonstrating the utility of microbial composition as a predictive biomarker for ICI efficacy (46, 50).

cmDNA has gained attention as a novel liquid biopsy biomarker. Its signatures differ between cancer patients and healthy individuals and are enriched in individuals with advanced disease (51). In several cancers, cmDNA levels and composition correlate with tumor burden, metastatic stage, and progression-free survival. Notably, bacterial DNA fragments derived from intra-tumoral or gut sources can be detected in the plasma, offering a non-invasive method for monitoring disease status and therapeutic response (50, 52). Beyond taxonomic signatures, microbial metabolites such as SCFAs, indoles, and bile acid derivatives are also being explored as functional biomarkers. These molecules modulate host immune tone, influence barrier integrity, and drive systemic inflammation factors that critically shape the tumor microenvironment and metastatic potential. Dysbiosis-induced shifts in microbial metabolite profiles are now being integrated into biomarker models to predict treatment outcomes (46, 53). Microbial markers have also been associated with resistance to therapy. In NSCLC and pancreatic cancer, the presence of *Gammaproteobacteria* within tumors or gut microbiota has been shown to degrade chemotherapeutic agents like gemcitabine, reducing efficacy and driving treatment failure. Conversely, antibiotic use before immunotherapy has been associated with poor outcomes, underscoring the predictive and prognostic value of microbiota integrity (24, 47).

Studies on non-gastrointestinal tumors further support the broader application of bacterial biomarkers. In breast, prostate, and lung cancers, bacterial taxa and their spatial distribution have shown prognostic value independent of traditional clinical parameters such as TNM staging or molecular subtype. Standardized protocols involving 16S rRNA sequencing, metagenomics, and qPCR are now enabling robust detection and validation of these microbial signatures (52, 54). The integration of microbial biomarkers into clinical decision making holds great promise for advancing precision oncology. Multi-omic platforms combining microbial data with genomic, transcriptomic, and immunologic profiles are being developed to stratify patients, monitor therapy response, and identify resistance mechanisms. These composite biomarkers may inform the timing and type of intervention, especially in immunotherapy-refractory cancers (48, 49, 51). In summary, microbial biomarkers represent a new frontier in oncology, offering insight into tumor-microbiome interactions that influence metastasis and therapy outcomes. As detection technologies and mechanistic understanding evolve, microbiome-informed diagnostics and prognostics will likely become integral to personalized cancer care.

## Conclusions and future perspectives

The microbiome has emerged as a critical yet underappreciated determinant of cancer metastasis, shaping the tumor microenvironment, immune responses, and therapeutic efficacy. Across metastatic settings, microbial communities interact with host cells via direct colonization, metabolite secretion, and modulation of intercellular signaling, thereby influencing every stage of tumor dissemination, from epithelial-mesenchymal transition to immune evasion and colonization of distant organs (4, 18, 26). Microbial signatures are not only markers but active participants in metastatic progression, reinforcing the notion that tumor-associated microbiota represent a dynamic and targetable component of cancer biology (9, 20).

One of the most transformative insights from recent studies is the realization that TRM are distinct from transiently associated taxa. These stable microbial inhabitants actively modulate oncogenic signaling, stromal remodeling, and therapeutic responses at both primary and metastatic sites. Moreover, TRM and their metabolites can influence chemoresistance and immunotherapy outcomes by altering drug metabolism, antigen presentation, and immune checkpoint activity (18, 48, 55). The integration of spatial, single-cell, and multi-omic technologies has provided the resolution needed to map microbial niches within tumors, unveiling cell-type-specific interactions that are crucial for personalized intervention (1, 37).

Despite the rapid expansion of microbiome-oncology research, several challenges persist. First, there is a need for rigorous standardization in sample processing, sequencing, and contamination control to ensure reproducibility across studies. Second, mechanistic validation of causal relationships remains limited and requires functional models that recapitulate microbial-tumor-immune interactions *in vivo*. Third, patient heterogeneity, including host genetics, diet, geography, and prior treatments, must be systematically accounted for to avoid confounding effects in microbial biomarker discovery (26, 50).

Looking ahead, several promising directions stand out. Microbial profiling is poised to become an essential component of cancer diagnostics and risk stratification. The development of non-invasive assays based on circulating microbial DNA, metabolomics, circadian rhythmicity, multi-omic data integration could enable real-time monitoring of metastatic progression and therapy responsiveness (20, 40, 56). In parallel, rational manipulation of the microbiota using engineered bacteria, bacteriophages, prebiotics, or fecal microbiota transplantation (FMT) offers novel therapeutic avenues for restoring immune competence and sensitizing tumors to immunotherapy (1, 4, 9).

Furthermore, clinical trials that incorporate microbial endpoints, either as primary outcomes or stratification variables, will be key to translating microbiome science into actionable oncology practices. The inclusion of microbial biomarkers into predictive frameworks, alongside genomics and immunoprofiling, has the potential to refine precision medicine and guide therapeutic decisions in metastatic disease (37, 48, 50). In conclusion, the intersection of microbiology and metastasis research opens a paradigm-shifting frontier in cancer biology. As our understanding of microbial-tumor-host crosstalk deepens, the microbiome will no longer be seen as a peripheral factor but as a central modulator of metastatic behavior and therapeutic response. Harnessing this knowledge may ultimately transform how we detect, monitor, and treat metastatic cancer.
